# Mathematical Modeling of Calcium Waves Induced by Mechanical Stimulation in Keratinocytes

**DOI:** 10.1371/journal.pone.0092650

**Published:** 2014-03-24

**Authors:** Yasuaki Kobayashi, Yumi Sanno, Akihiko Sakai, Yusuke Sawabu, Moe Tsutsumi, Makiko Goto, Hiroyuki Kitahata, Satoshi Nakata, Junichi Kumamoto, Mitsuhiro Denda, Masaharu Nagayama

**Affiliations:** 1 Research Institute for Electronic Science, Hokkaido University, Sapporo, Japan; 2 CREST, Japan Science and Technology Agency, Tokyo, Japan; 3 Graduate School of Science and Technology, Kanazawa University, Kanazawa, Japan; 4 Shiseido Research Center, Shiseido Co., Ltd., Yokohama, Japan; 5 Department of Physics, Graduate School of Science, Chiba University, Chiba, Japan; 6 Graduate School of Science, Hiroshima University, Higashi-Hiroshima, Japan; Fondazione Edmund Mach, Research and Innovation Centre, Italy

## Abstract

Recent studies have shown that the behavior of calcium in the epidermis is closely related to the conditions of the skin, especially the differentiation of the epidermal keratinocytes and the permeability barrier function, and therefore a correct understanding of the calcium dynamics is important in explaining epidermal homeostasis. Here we report on experimental observations of in vitro calcium waves in keratinocytes induced by mechanical stimulation, and present a mathematical model that can describe the experimentally observed wave behavior that includes finite-range wave propagation and a ring-shaped pattern. A mechanism of the ring formation hypothesized by our model may be related to similar calcium propagation patterns observed during the wound healing process in the epidermis. We discuss a possible extension of our model that may serve as a tool for investigating the mechanisms of various skin diseases.

## Introduction

The barrier function of the skin is maintained by adjusting its state while sensing changes in chemical and physical stimuli received from internal and external environments [1]. In other words, keratinocytes are sensitive to such stimuli and are able to recover the damaged epidermal barrier. Recent studies have revealed that the calcium dynamics in the epidermal keratinocytes is strongly associated with cutaneous homeostasis: modulations in epidermal calcium concentration coordinately regulate events late in the epidermal differentiation that together form the barrier [2]. For example, Menon et al. [3] have demonstrated that alteration of the calcium gradient in the epidermis affects the exocytosis of the epidermal lamellar bodies. It is also known that the concentration of calcium is highest in the uppermost region of the epidermis (the epidermal granular layer) in healthy normal skin, and that the calcium gradient disappears immediately after barrier disruption [4], [5]. Moreover, abnormal calcium gradients in the epidermis have been observed in a variety of skin diseases [6], and the mutation of the calcium pump or gap junctions is known to induce genetic skin diseases [7]–[9]. In addition, it has been reported that information regarding the stimuli, damage status, and the skin pathology are reflected in the features of calcium wave propagation and distribution in cultured keratinocytes [10], [11]. Therefore, understanding the mechanisms of the dynamical behavior of the calcium distribution in the epidermal keratinocytes should provide us with clues regarding the possible treatment of various skin diseases.

Although individual phenomena regarding the spatio-temporal dynamics of calcium ions have been investigated, the relationships between these phenomena, and how they are related to the epidermal structure and the barrier function have not yet been clarified. Conventional research on dermatology usually adopts the methodology of biochemistry or molecular biology, and these approaches enable us to discuss the detailed relationship between the chemicals and the molecular functions. On the other hand, formulating a mathematical model for describing the global behavior of calcium waves enables us to discuss the functions of calcium-wave related phenomena within the cells, and should even show several guidelines for directing future dermatological research. Such a model would be incorporated into the mathematical model of the epidermis, where the interaction of the structure and the calcium dynamics could be simulated. Therefore, a mathematical approach may help us to understand not only individual functions but also the whole system as a hierarchical structure.

**Figure 1:**
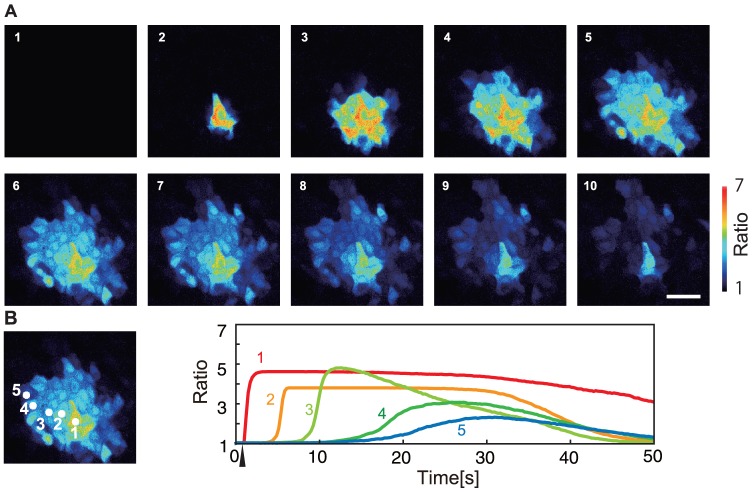
Experimental results on the calcium wave propagation induced by a mechanical stimulation to one cell. (A) Snapshots with an interval of 4.86 s. The scale bar is 100 μm. The ratiometric images (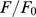) are shown in false color, where blue, green, yellow, and red indicate the increase in intracellular calcium concentration, in this order (see the scale bar on the right). (B) Time change in the calcium concentration is shown in each cell depicted in the left figure. The time when mechanical stimulus was applied is indicated by an upper arrowhead.

Calcium waves have been mathematically understood as traveling waves on excitable media [12]–[14]. A traveling wave on excitable media, once triggered by a sufficiently strong stimulus, propagates infinitely by exciting neighboring regions continuously. This excitable media picture, however, is insufficient to reproduce the features of calcium waves in keratinocytes; calcium waves in keratinocytes do not propagate infinitely but stop within a finite area. We have already obtained important results on the in vitro observation of calcium waves induced by mechanical stimulation on cultured keratinocytes [15]. The experimental results reproduced under a different condition (see Materials and Methods) is shown in Fig. 1. The concentration of calcium ions in the stimulated cell increases, followed by an increase in calcium concentration in the neighboring cells. However, the calcium wave propagates only in a restricted region, up to about five cell diameters away from the stimulated cell. Recently, Warren et al. has proposed a mathematical model of ATP-mediated calcium waves in mammalian airway epithelium with many internal variables and succeeded in reproducing finite range propagation of calcium waves [16]. Also, Edwards and Gibson introduced a similar model to explain calcium waves in astrocytes [17]. In contrast to their study, we need a simpler model for calcium waves in keratinocytes, in order to investigate the dynamical behavior of the in vivo three-dimensional structure of the epidermis, especially the barrier function of it.

In this paper, using known biochemical and dermatological results, we introduce a mathematical model that can account for the behavior of calcium waves in keratinocytes, especially a ring-shaped pattern recently observed by Tsutsumi et al. [18]. In order to compare our numerical simulations with experimental data, previous experiments are reproduced and the calcium levels are measured under several conditions. We discuss the relation of the ring pattern to skin diseases, and a possible extension to a mathematical model of the epidermal structure.

## The Mathematical Model

Several mathematical models, such as the “one-pool” and “two-pool” models, have been proposed for describing intracellular calcium waves. In the former model, it is assumed that calcium ions and IP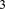 both affect a single type of calcium ion pool [19]–[22]. In the latter model, it is assumed that there are two types of calcium ion pools in each cell, where one pool has receptors for calcium ions and the other has receptors for IP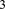 [13], [23]. The one-pool model has been often used in recent studies, due to its ability to reproduce several experimental results. We adopt the “one-pool model” proposed by Atri et al. [23] as the basis of our model, to which the following assumptions are added: (i) ATP diffuses in a culture solution. (ii) ATP can be detected by ATP-receptors on cell membranes, and IP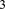 is synthesized inside each cell [24], [25]. (iii) IP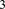 and calcium ions inside cells can travel through gap junctions and excite neighboring cells. (iv) Gap junctions close when the Ca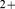 gradient between cells becomes large.

Based on these assumptions, we propose a mathematical model consisting of ordinary and partial differential equations for the following variables; 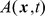 (the ATP concentration in a culture solution), 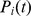 (the IP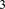 concentration in the 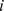
th cell), 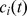 (the calcium ion concentration in the 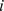
th cell), 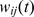 (the activity of the gap-junctions between cells 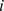 and 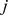), and 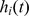 (the inactivation factor of the 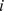
th cell, which is defined in the “one-pool model”).

We consider a two dimensional space, where 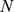 cells with radius 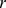 are randomly distributed. The center of the 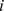
th cell is located at 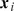 and any two cells are connected through gap-junctions when their distance is less than 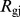. To calculate the ATP dynamics, we utilize the bi-domain method: we treat the space as cell-free when calculating the concentration field 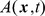, and when calculating intracellular dynamics of the cell 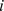, the value of the surrounding ATP concentration is represented by 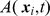. The mathematical model including all these features is as follows:
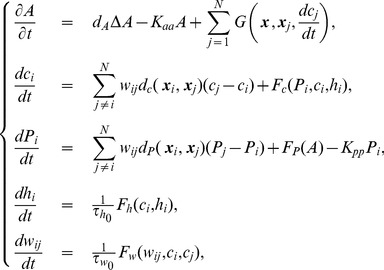
(1)where
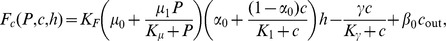





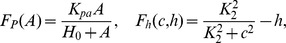





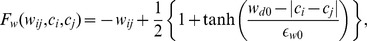





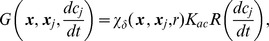





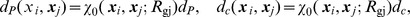





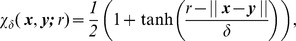





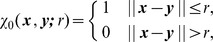





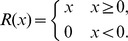



**Figure 2:**
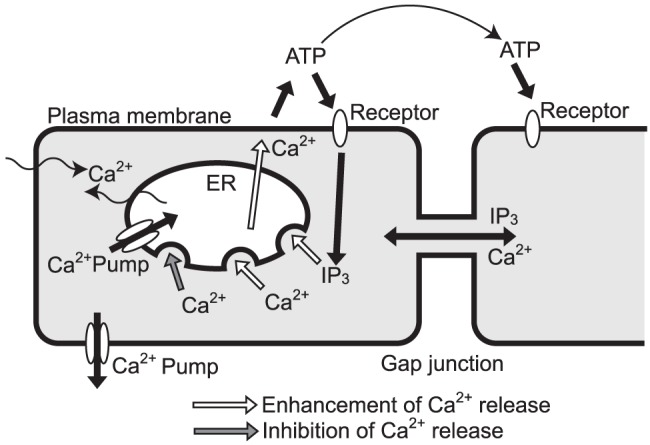
Schematic illustration of the relationship among concentrations of Ca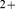, IP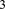, and ATP. White and gray arrows denote the enhancement and inhibition of Ca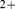 release, respectively.

The relationships among the concentrations of calcium ions, IP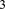, ATP, etc., are illustrated in Fig. 2.

The effect of the mechanical stimulation is described as an increase in the concentration of calcium ion, in the stimulated cell. Since it is known that the stimulated cell releases ATP to the culture solution [15], [24], and that ATP release is observed even without the calcium excitation [10], the extracellular ATP concentration, near the stimulated cell is also increased independently of the calcium excitation. The mechanical stimulus is represented by the stimulus function 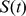. We assume that the effect of stimulation appears in the model equations as the time derivative of 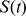, not 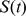 itself, reflecting the fact that ATP release does not continue under continuous stimuli. By the same token, the function 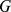, the ATP release due to the calcium excitation, depends not on the calcium concentration itself but on the time derivative of it. Also, following a recent experimental result [18], we further assume that when a cell is broken by a strong stimulus, a stimulant 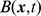 is released that induces the influx of extracellular Ca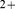 through calcium channels on the plasma membrane. These features are introduced to our mathematical model to reproduce the experimental findings of the mechanical stimulation:
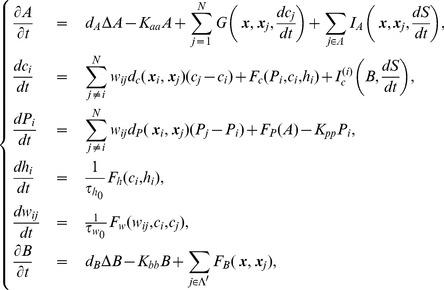
(2)where
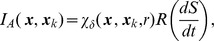





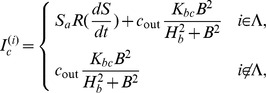





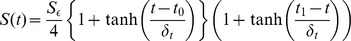





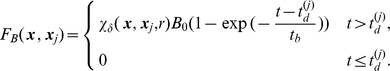
Here 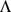 and 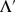 denote the set of mechanically stimulated and broken cells, respectively, and 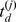 indicates the time that cell 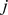 is broken. All the other parameters not mentioned above are explained in Table 1.

**Table 1:** List of parameters.

parameter	meaning
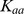	degradation rate of ATP
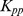	degradation rate of IP 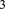
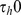	typical timescale of the inactivation factor
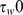	typical timescale of the gap-junction activity
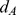	diffusion coefficient of ATP
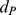	diffusion rate of IP 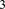 through gap-junctions
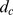	diffusion rate of Ca 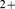 through gap-junctions
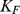	maximum Ca 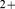 flux through IP 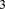 receptors
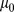	probability of the spontaneous activation at the IP 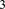 binding domain
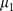	maximum probability of the activation at the IP 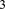 binding domain due to IP 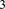
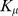	typical value of IP 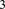 at which the activation by IP 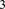 becomes effective
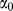	probability of the spontaneous activation at the first Ca 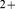 binding domain
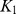	typical value of IP 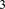 at which the activation by Ca 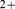 becomes effective
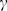	maximum Ca 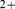 flux due to Ca 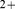 pumping out of the cytosol
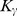	typical value of Ca 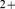 at which Ca 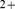 pumping becomes effective
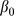	coefficient of Ca 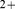 leaking into the cytosol from the extracellular Ca 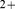
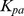	maximum rate of the IP 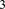 production due to ATP
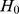	typical value of the ATP at which the IP 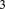 production becomes effective
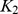	typical value of Ca 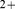 at which activation of the second Ca 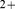 binding domain becomes effective
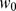	threshold of Ca 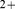 concentration difference for gap-junction closing
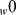	sensitivity for gap-junction closing
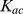	coefficient of ATP production due to Ca 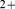 increase
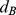	diffusion coefficient of the stimulant
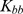	degradation rate of the stimulant
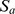	coefficient of the flux due to mechanical stimulation
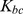	coefficient of the maximum flux due to the stimulant
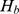	typical value of the stimulant at which the stimulant-induced Ca 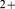 flux becomes effective
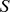	maximum strength of mechanical stimulation
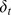	width of the transition layer between the on and off state of the stimulation
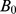	saturated value of the stimulant release rate
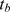	typical timescale of the stimulant release

When a stimulus is too strong and the Ca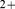 level reaches a certain threshold 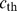, we consider that the cell is broken, and set 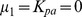 for that cell, so that the Ca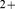 and IP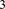 levels relax to stationary states. At this point the stimulant 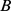 is released. We also assume that the stimulant 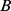 affects the Ca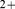 level only when the extracellular calcium 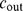 exists [18].

We now turn to the normalization of our model equations. Let us introduce the following dimensionless variables:
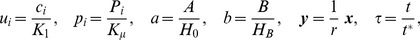
(3)where 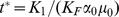. Then the model equations are rewritten accordingly:
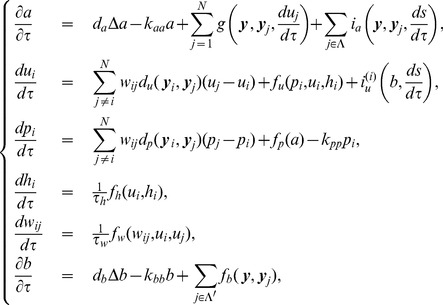
(4)where
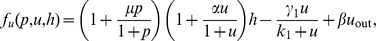





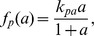





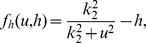





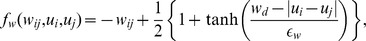





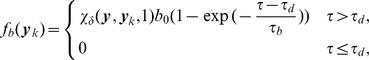





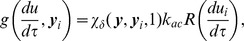





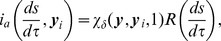





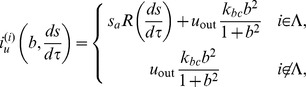





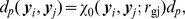





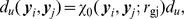





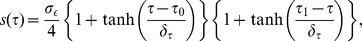



and the parameters are rescaled as 
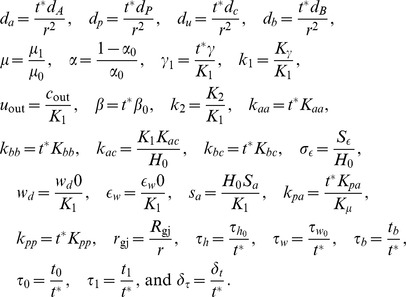


It is noted that the effect of the diffusion of calcium ions is small, since the calcium ions can react with many kinds of proteins in the cells before diffusing through a gap junction. Hence we assume 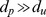.

We set the system size 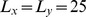. The equations for 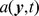 and 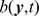 are solved using the ADI method with a grid size of 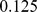 under Neumann boundary conditions. The remaining equations are solved using an explicit Euler scheme with a time step of 0.001.

The initial conditions are assigned in such a way that all variables are in a steady state: 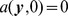, 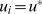, 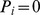, 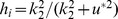, 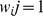, and 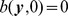, where 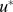 is the solution to 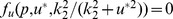. The threshold 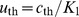 is set to 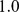.

Unless otherwise noted, the remaining parameters are set with the following values: 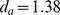, 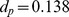, 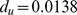, 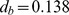, 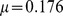, 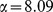, 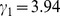, 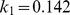, 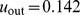, 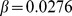, 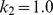, 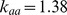, 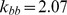, 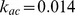, 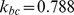, 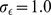, 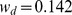, 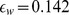, 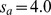, 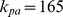, 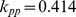, 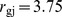, 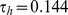, 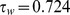, 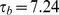, 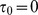, 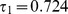, and 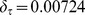. For these parameters we obtain 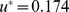.

## Results and Discussion

**Figure 3:**
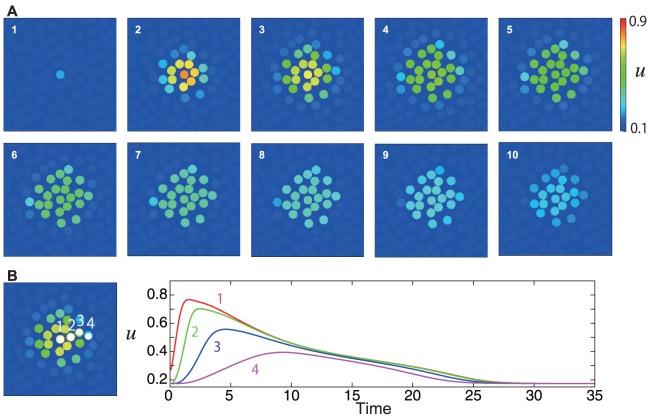
Numerical results of the mechanical stimulation. Stimulation is given to the cell located at the center at 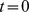. (A) Snapshots every 2 time units are shown. (B) Changes in the calcium concentration of the cells are specified in the left figure.

In the following numerical simulations, we apply a mechanical stimulation to a single cell which is located at the center of the domain. We first perform a simulation with a modest stimulation strength, 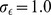, which implies that the stimulated cell is not broken and therefore that the stimulant 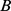 is not released. The results of the numerical simulation are shown in Fig. 3 A, which reproduces the experimentally observed finite-range wave propagation: the calcium wave only reaches a distance of several cells from the stimulated one.

Note that the finite-range wave propagation does not stem from a discrete nature of the cells, as is often found in discrete excitable media [26]: we have numerically checked that even when we take the continuous limit for all discrete variables, we still found finite range propagations.

### The Effect of ATP-breaker and gap-junction blocker

**Figure 4:**
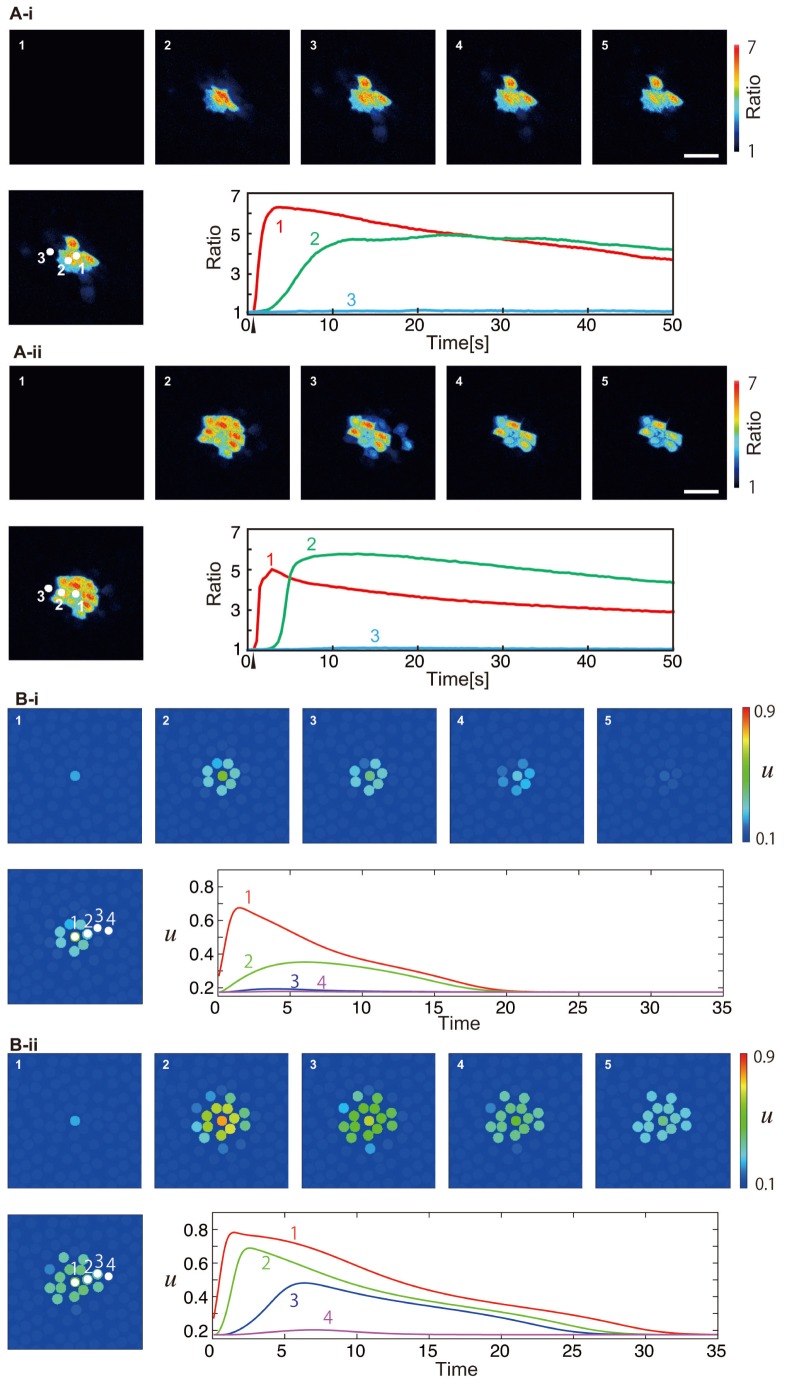
Experimental and numerical results with the addition of ATP-degrading enzyme and gap-junction-blocking reagent. (A-i) Experimental results with the addition of ATP-degrading enzyme (apyrase). In the upper figures, snapshots are shown with an interval of 10.7 s. The scale bars are 100 μm. The ratiometric images (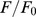) are shown in false color, where blue, green, yellow, and red indicate increasing intracellular calcium concentration, in this order (see the scale bar on the right). In the figure below, change in the calcium concentration throughout time is shown for each cell depicted in the left figure. The time when the mechanical stimulus was applied is indicated by an upper arrowhead. (A-ii) Experimental results with the addition of gap-junction-blocking reagent (octanol). (B) The numerical results corresponding to the addition of (B-i) ATP degrading enzyme and (B-ii) gap-junction-blocking reagent. The parameters are the same as those used for Fig. 1 except for (B-i) 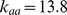, and (B-ii) 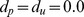. Snapshots every 5 time units are shown. Below, change in 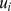 is shown throughout time in each cell depicted in the left figure.

To confirm the validity of our model, we compare the experimental and numerical results under the condition of an additional ATP-degrading enzyme and a gap-junction-blocking reagent. The experimental results, which are a reproduction of the previous work [15] under a different condition, are shown in Fig. 4, A-i and A-ii. We note that the area where the calcium wave propagates is smaller in comparison with the results shown in Fig. 1. In the numerical simulation, the addition of the ATP-degrading enzyme corresponds to an increased 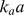, and the gap-junction-blocking reagent corresponds to smaller 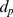 and 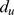. The results of numerical simulation for such conditions are shown in Fig. 4, B-i and B-ii. The experimental results correspond well to the numerical ones.

### Dependency on the strength of the mechanical stimulation

**Figure 5:**
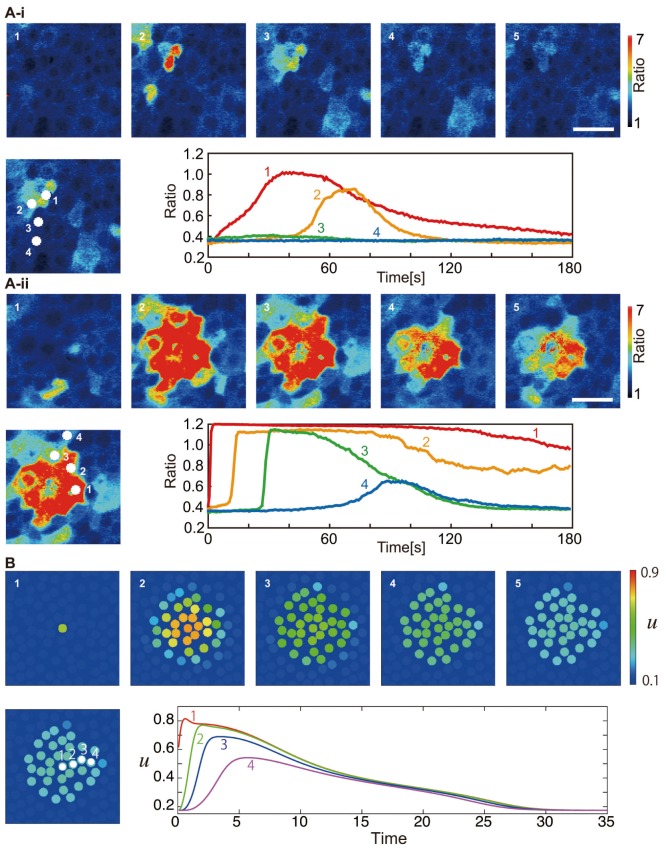
Comparison between (A) experimental and (B) numerical results when the strength of the stimulus is varied. (A) In the experiments, the pressure imparted on the cell was (A-i) 50 hPa and (A-ii) 100 hPa. In the upper figures in (A), snapshots are shown with the time intervals 40.0 s. The scale bars are 50 µ

We have also compared an experimental and numerical results where the strength of the mechanical stimulation is varied. The experimental results, which are a reproduction of [27] under a different condition, are shown in Fig. 5 A, where we observe that the calcium wave propagates further when the stimulation is stronger. In the corresponding numerical simulation, we use a stronger stimulus 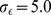, which is still weak so that the stimulant 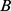 is not released. The numerical results are shown in Fig. 5 B, which reproduces the expansion of the propagation range observed in the experiment.

m. The ratiometric images (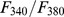) are shown in false color, where blue, green, yellow, and red indicate increasing intracellular calcium concentration, in this order (see the scale bar on the right). Below, change in the calcium concentration is shown throughout time in each cell depicted in the left figure. The origin was set to be the time that the mechanical stimulus was applied. (B) Numerical results with the same settings as that of Fig. 3, except that the stimulus is five times stronger, 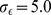.

### Ring pattern

**Figure 6:**
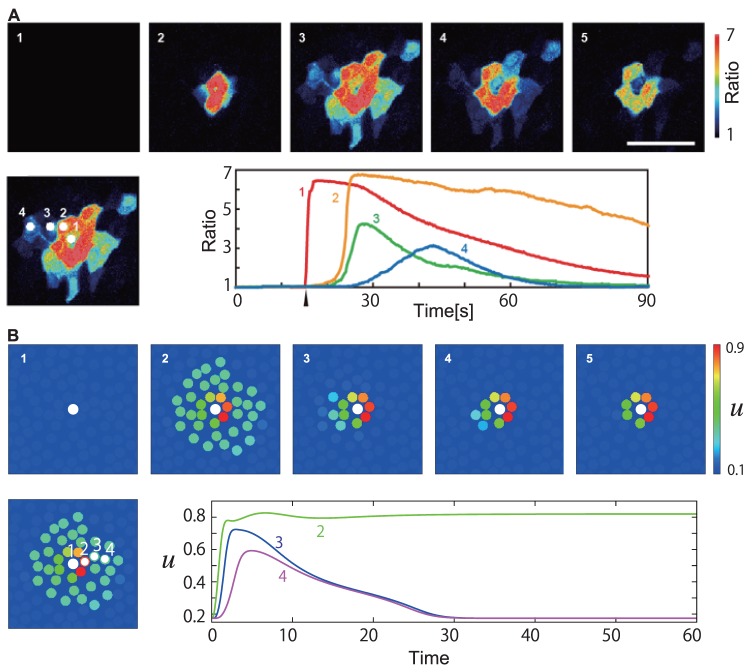
Ring patterns when a cell is broken by a strong mechanical stimulation. In experiment (A), a localized high-calcium-concentration region was observed around the broken cell. Snapshots are shown every 19.0 s. The scale bar is 100 μm. The ratiometric images (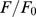) are shown in false color, where blue, green, yellow, and red indicate the increase in intracellular calcium concentration, in this order (see the scale bar on the right). Changes in the calcium concentrations are shown throughout time in the bottom right for the cells numbered in the left panel. The time that the mechanical stimulus was applied is indicated by an upper arrowhead. (B) Numerical results with the same settings as that of Fig. 3, except that the stimulus is ten times stronger, 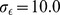. Snapshots every 15 time units are shown. The calcium level of cell 1, which immediately crosses 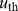 after stimulation, is not shown.

It has recently been reported that a strong mechanical stimulus, enough to break the cell, causes a ring-like Ca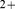 localized pattern around the broken cell [18]. This experimental observation can also be reproduced by our model, by using an even stronger stimulus 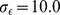. The numerical results are shown in Fig. 6. We also reproduced the experiment [18] under a different experimental condition and measured the calcium levels for comparison with the simulation data (also shown in Fig. 6). In this case the Ca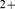 level of the stimulated cell immediately crosses the threshold 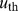 and the cell is broken, leading to the release of the stimulant 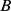. The gap junctions connecting the broken cell to the neighbors immediately close due to the dynamics of 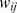, so that the influx of Ca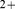 from the broken cell is blocked. Also, since the gap junctions connecting the excited cells to the neighbors are closed for the same reason, the effect of excitation reaches only one cell diameter away, resulting in a ring pattern.

We have confirmed that the ring pattern is not formed when we set the extracellular calcium to zero, 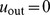. This means the stimulant 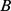 cannot affect the intracellular calcium dynamics. This is in accord with the experiment [18], where ring patterns were not observed when the mechanical stimulation was performed in the calcium-free medium.

### General characteristics of calcium wave propagation in keratinocytes

From these results, it is confirmed that our mathematical model qualitatively reproduces the features of calcium wave propagation in cultured keratinocytes. Our model suggests that the essence of calcium wave propagation is ATP diffusion and transportation of IP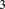 through gap junctions. The calcium waves created in our model propagate only within a limited area, in marked contrast to excitable waves in nerve cells, which is often represented by the Hodgkin-Huxley model [28] and the FitzHugh-Nagumo model [29], [30]. In our model, the excited region expands not infinitely but finitely, and the excited region comes back to the steady state. Thus, the excited region looks like a disk, as is observed in the experiments.

We speculate that the finite range wave propagation, and especially the ring pattern formation might be used in vivo to define abnormal and damaged sites: when the damaged skin is repaired, information on the location of the damaged site must be detected, and the localized calcium excitation would serve as such a signal. We also hypothesize that the ring pattern-generating mechanism is responsible for the calcium localization beneath the stratum corneum in the epidermis: In the process of cornification, keratinocytes, undergoing denucleation, would release stimulants as we assumed, which would cause the experimentally observed localized calcium excitations just below the cornified layer, which then induces the differentiation into cornified cells. Hence we think that our calcium dynamics model can be incorporated into an epidermal cell model to simulate epidermal homeostasis. Such a model can be used, for example, to study the importance of calcium dynamics for the structural stability of the epidermis.

Abnormal distribution of calcium in the epidermis is observed in atopic dermatitis, psoriasis [6], and the aged skin [31]. These skin problems can be characterized by the abnormal barrier function [1]. Since calcium distribution is strongly associated with the permeability barrier homeostasis [2], the extended model could also be used as a simulator of these skin diseases.

## Materials and Methods

### Cells and cell culture

Normal human epithelial keratinocytes were purchased from Kurabo (Osaka, Japan) and cultured in EPILIFE-KG2 (Kurabo). Keratinocytes were seeded onto collagen-coated glass coverslips (Matsunami, Osaka, Japan) and used within 5 days. Keratinocytes were grown to approximately 100%-120% confluency in the presence of 0.06 mM Ca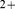, and this was switched to 1.8 mM Ca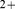 24 h before experiments.

### Measurement of intracellular calcium ions

Changes in intracellular calcium concentration in individual cells were measured with Fluo-4 AM (Molecular Probes Inc., OR, USA) for Figs. 1, 4 A and 6 A, and Fura-2 AM (Molecular Probes Inc., OR, USA) for Fig. 5 A. Cells were loaded with 5 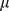
M Fluo-4 AM at 37

C for 40 min. After loading, cultures were rinsed in BSS containing (in mM): NaCl 150, KCl 5, CaCl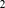 1.8, MgCl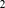 1.2, HEPES 25, and D-glucose 10 (pH 7.4), and incubated for a further 10 min at room temperature to allow de-esterification of the loaded dye. The coverslip was mounted on an inverted epifluorescence microscope (Eclipse Ti, Nikon, Tokyo, Japan). The image data were obtained with a high-sensitivity CCD camera (ORCA-R2, Hamamatsu Photonics, Hamamatsu, Japan) under the control of analyzing system (AQUACOSMOS/RATIO, Hamamatsu Photonics). The fluorescence signals of Fluo-4 AM (for Figs. 1, 4 A and 6 A) were indicated by ratiometric images (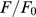), where 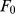 represents the initial level of fluorescence and 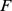 is the fluorescence recorded at different time points during the experiment. On the other hand, the fluorescence signals of Fura-2 AM (for Fig. 5 A) were represented as the ratio of fluorescence intensities at 340 and 380 nm (emission wavelength was 510 nm), i.e., 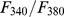, where 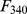 and 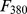 are the fluorescence intensities at 340 and 380 nm, respectively.

### Mechanical stimulation

A glass micropipette was made by pulling a glass capillary (Sutter Instrument, CA, USA) with a puller and mounted on a micromanipulator (Narishige, Tokyo, Japan). The micropipette was placed over a single cell and mechanical stimulation was applied to the cell by lowering the micropipette onto the surface of the cell, and then (i) rapidly returning it to its original position (Fig. 1), or (ii) keeping it in that position for an interval of time (Fig. 6). In case (i), if the stimulated cell shows any damage like dye leakage or abnormal morphology, or increase in fluorescence in the second stimulation, the experiment was eliminated. The same experiments were performed under the condition where an ATP degrading enzyme (apyrase) or gap-junction-blocking reagent (octanol) was added to the culture solution (Fig. 4 A). Apyrase and octanol were purchased from Sigma-Aldrich (St. Louis, MO, USA).

Hydraulic pressure stimulation was performed using a FemtoJet microinjector (Eppendorf, Hamburg, Germany) in BSS. A glass microtube was pulled from glass capillary and was filled with BSS or calcium-free BSS. It was connected with the microinjector and its tip was placed just over the cell. Pressure and injection time were controlled by the microinjector. First, the fluorescence intensity of the unstimulated cell was measured, and then the cell was stimulated with hydraulic pressure for 1 second. The fluorescence intensity was measured for more than 5 minutes (Fig. 5 A).

## Conclusions

We have proposed a mathematical model for calcium wave propagation in keratinocytes considering the concentration of ATP as an extension of the “one-pool model” suggested previously. Using our model, we have reproduced experimental results of the calcium wave propagation in keratinocytes with the addition of an ATP-degrading enzyme or a gap-junction-blocking reagent, and achieved numerical results corresponding to the experimental observations where the strength of the mechanical stimulation is varied. By assuming the release of a stimulant from a broken cell, an experimentally observed ring pattern has also been reproduced. Through making our model, we suggest that the finite wave propagation is important for calcium waves in keratinocytes, i.e., calcium waves in keratinocytes do not propagate like traveling pulses, as in nerve cells, but propagate finitely while remaining in the shape of a disk. Although microscopic mechanism of the calcium propagation in the epidermis is still unknown, our model is expected to be used for studying of the role of calcium dynamics for the structural stability of the epidermis by incorporating it into epidermal cell models, which will be of great use for the cure of various skin diseases.
